# Context-dependent miRNA regulation in intervertebral disc degeneration: an IDD-focused dynamic network framework for stage-specific therapeutics

**DOI:** 10.3389/fcell.2026.1844246

**Published:** 2026-06-11

**Authors:** Yanchao Gu, Ling Zhu, Wang Chen, Xianghu Zhao, Zhanghua Li

**Affiliations:** 1 College of Sports Medicine, Wuhan Sports University, Wuhan, China; 2 Wuhan Sports University, Wuhan, China; 3 Department of Orthopaedics, Wuhan Third Hospital, Tongren Hospital of Wuhan University, Wuhan, China

**Keywords:** ceRNA network, context-dependent regulation, engineered exosomes, intervertebral disc degeneration, low back pain, mechanobiology, microRNA

## Abstract

Intervertebral disc degeneration (IDD) is a progressive, mechanically regulated, and inflammation-associated disease in which microRNAs (miRNAs) participate in extracellular matrix remodeling, cell death, inflammatory signaling, and intercellular communication. However, the reported functions of individual miRNAs in IDD are often inconsistent across studies, with the same miRNA being associated with protective, neutral, or pro-degenerative outcomes under different experimental or pathological conditions. This inconsistency highlights a central knowledge gap: whether miRNA function in IDD is determined primarily by intrinsic molecular identity or by the dynamic pathological context in which the miRNA is embedded. In this Hypothesis and Theory article, we propose the Context-Dependent miRNA Switching Model (CDMSM), an IDD-focused conceptual framework in which degeneration stage, mechanical loading, inflammatory intensity, oxidative stress, extracellular matrix status, and ceRNA-network remodeling jointly reshape miRNA-target interactions and thereby alter biological output. The model predicts that selected miRNAs may display stage-dependent target bias, altered effective concentration due to ceRNA competition, and different therapeutic effects across early, intermediate, and chronic/late-stage IDD. We further discuss how CDMSM may guide stage-specific miRNA therapeutics, including engineered exosomes, miRNA inhibitors, and microenvironment-responsive biomaterials. Finally, we outline experimental strategies required to test the model, including longitudinal animal models, controlled mechanobiology systems, single-cell and spatial transcriptomics, and functional perturbation of miRNA-target networks.

## 1 Introduction

IDD is the primary pathological basis of low back pain (LBP) and a major cause of patient disability, posing a substantial threat to society and healthcare systems (Mohd Isa et al., 2022). Immune microenvironment alterations are also increasingly recognized as contributors to IDD pathophysiology (Ren et al., 2025). The traditional view considered IDD a passive degenerative disease associated with aging; however, increasing evidence indicates that it is an active pathological process driven jointly by multiple factors, including genetic background, mechanical loading, inflammation, the microenvironment, and metabolism (Ohnishi et al., 2022). During this process, dynamic changes in the phenotype of nucleus pulposus (NP) cells are particularly critical. As degeneration progresses, NP cells gradually transition from homeostasis to programmed cell death pathways, such as senescence, apoptosis, pyroptosis, and even ferroptosis (Shnayder et al., 2023). Concurrently, inflammatory cytokines (e.g., IL-1β, TNF-α) and matrix-degrading enzymes (matrix metalloproteinases [MMPs], a disintegrin and metalloproteinase with thrombospondin motifs [ADAMTSs]) form a continuously amplifying positive feedback loop, accelerating ECM destruction (Yang et al., 2021). Therefore, the pathological process of IDD involves the intertwining of multiple factors, making conventional interventions targeting a single molecule often ineffective.

In this article, we employ the term CDMSM as an IDD-focused working framework to bridge the gap between general miRNA biology and disc-specific pathology. Context-dependent and even apparently opposing miRNA functions have already been reported in other fields, including cancer, immune regulation, and fibrosis. The intended contribution of this review is to apply this broader principle systematically to IDD, where degeneration stage, mechanical loading, inflammation, hypoxia, acidity, ECM remodeling, and cellular heterogeneity evolve together.

MicroRNAs are a class of endogenous non-coding RNAs, approximately 22 nucleotides in length, that regulate gene expression post-transcriptionally by binding to the 3′-untranslated regions of target mRNAs (O’Brien et al., 2018). Because a single miRNA can simultaneously target multiple genes, and a single gene may be co-regulated by multiple miRNAs, this “many-to-many” characteristic makes miRNAs crucial regulatory nodes within cellular signaling networks. Studies have demonstrated that miRNAs participate in various key processes associated with IDD, including cell proliferation and apoptosis (Chen et al., 2025), ECM synthesis and degradation (Liu et al., 2017), inflammatory response regulation (Li et al., 2022), and exosome-mediated intercellular communication (Wang et al., 2024). However, we have observed that descriptions of the same miRNA’s function across different studies are not always consistent, a phenomenon that is particularly prominent in the field of IDD research and has been summarized in comprehensive reviews of miRNAs in IDD (Cazzanelli and Wuertz-Kozak, 2020).

IDD is particularly suitable for such an analysis because the disc microenvironment is mechanically loaded, poorly vascularized, relatively hypoxic and acidic, and progressively remodeled during degeneration. Thus, the same miRNA may encounter different target abundance, ceRNA competition, inflammatory tone, matrix composition, and cellular-subpopulation structure at different disease stages. This biological setting provides a rationale for considering IDD within the broader spectrum of diseases in which miRNA functions are context-sensitive.

A systematic bibliometric analysis has already emphasized that inconsistencies in IDD-related miRNA studies may arise from differences in research models, stimulation methods, and expression levels (Chen et al., 2023). Building on this important observation, we propose CDMSM as a structured, hypothesis-generating framework to organize IDD-specific miRNA evidence. Under this framework, miRNA function in IDD is interpreted according to three interrelated principles: (1) stage dependence, (2) network dependence, particularly ceRNA remodeling, and (3) microenvironmental threshold effects. These principles do not exclude methodological explanations; rather, they provide testable biological hypotheses that should be evaluated only after major experimental variables are carefully controlled.

**FIGURE 1:**
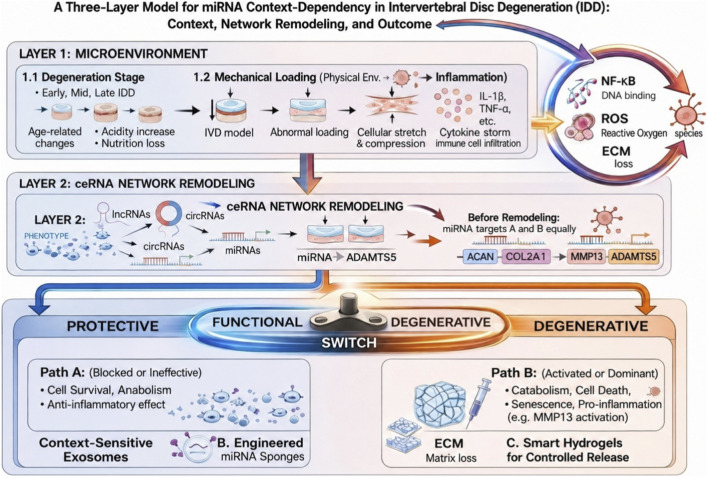
Three-layer conceptual framework of context-dependent miRNA regulation in intervertebral disc degeneration. Microenvironmental factors, including degeneration stage, mechanical loading, and inflammatory signaling, reshape ceRNA regulatory networks in disc cells. Network remodeling alters miRNA–target interactions and leads to target gene switching, thereby producing distinct biological outcomes ranging from protective ECM maintenance to pathological degeneration. These insights highlight the importance of context-matched therapeutic strategies, including engineered exosomes, miRNA sponges, and smart biomaterial-based delivery systems.

From a translational standpoint, comparative and veterinary studies can help contextualize CDMSM. Naturally occurring canine IDD has been proposed as a clinically relevant model for human disc degeneration (Bergknut et al., 2012), while species differences in nucleus pulposus cell morphology and notochordal-cell persistence may influence disc-cell phenotype and miRNA-network behavior (Hunter et al., 2004). These observations support the need to test whether context-dependent miRNA regulation is conserved across species, mechanical environments, and degeneration tempos (Figure 1).

## 2 Core pathological mechanisms mediated by miRNAs

### 2.1 NP cell phenotypic homeostasis

miR-494 serves as a classic example elucidating the context dependence of miRNA functions. In an *in vitro* model of acute inflammatory stimulation, it was found that inhibiting miR-494 could attenuate TNF-α-induced NP cell apoptosis, indicating that miR-494 may be associated with pro-apoptotic effects in this stress context (Wang et al., 2015). However, another study found that miR-494 is also significantly upregulated in severely degenerated samples, exhibiting a positive correlation with decreased SOX9 expression and enhanced matrix degradation (Kang et al., 2017). Therefore, it is evident that the role of miR-494 differs across various stages of disc degeneration. It should also be noted that the aforementioned evidence is primarily derived from cross-sectional observations across different studies and does not directly prove that miR-494 undergoes a definitive temporal functional transition within the same degenerative process. Thus, a more plausible explanation is that the biological effects of miRNAs are largely influenced by the specific pathological environment.

Taken together, these observations support CDMSM by suggesting that miRNA output in NP cells may depend on the phenotypic state of the cell and the severity of the degenerative microenvironment. Rather than acting as fixed protective or pathogenic factors, miRNAs may produce different outcomes when the cellular background shifts from adaptive stress response toward apoptosis, senescence, or matrix-catabolic remodeling. However, because the available evidence is largely cross-sectional, this interpretation should be regarded as hypothesis-generating (Wang et al., 2015; Kang et al., 2017).

### 2.2 ECM metabolism

Literature reports indicate that miR-365 is frequently closely associated with the body’s mechanical microenvironment and is considered a typical mechanosensitive miRNA (Zheng et al., 2019). Under cyclic pressure conditions within the physiological range (approximately 0.5–2 MPa), miR-365 helps maintain disc matrix homeostasis, whereas under persistent high-intensity hydrostatic pressure (>3 MPa) or pathological loading, miR-365 accelerates degeneration (Chan et al., 2011). Integrating recent reports on molecular mechanisms, such as under basal or moderate stress conditions, miR-365 can effectively inhibit NP cell apoptosis and maintain the balance of ECM synthesis and metabolism by directly targeting and regulating EFNA3 (Jiang et al., 2024). When encountering pathological-level mechanical overload, the expression level of miR-365 and its target gene network are highly susceptible to context-dependent remodeling, thereby altering or even reversing its protective effects on the ECM.

For ECM-related endpoints, the biological interpretation of a miRNA should include both anabolic and catabolic readouts. Aggrecan (ACAN), type II collagen (COL2A1), and SOX9 represent core markers of matrix preservation, whereas MMP3, MMP13, ADAMTS4, and ADAMTS5 reflect matrix degradation and aggrecanolysis. From the perspective of CDMSM, a miRNA that supports ACAN/COL2A1/SOX9-associated matrix maintenance under physiological or moderate loading may not exert the same network output under pathological overload, where inflammatory signaling, MMP/ADAMTS activity, and altered target accessibility dominate (Ohnishi et al., 2022; Jiang et al., 2024).

### 2.3 Inflammation and oxidative stress microenvironment

Studies have found that in IDD-related inflammatory models, the expression of miR-146a typically increases along with the upregulation of IL-1β or TNF-α, and it can inhibit the NF-κB signaling pathway by targeting TRAF6 and IRAK1, thereby mitigating the inflammatory cascade amplification and, to a certain extent, alleviating ECM degradation and cell apoptosis (Lv et al., 2017). However, as the degenerative microenvironment continues to deteriorate, intracellular oxidative stress and mitochondrial dysfunction progressively become key pathological features; miR-146a-5p then targets antioxidant-related molecules, such as superoxide dismutase 2 (SOD2), exacerbating oxidative stress levels and the degree of cellular damage (Gao et al., 2025). This finding suggests that the role of miR-146a may be influenced by the status of multiple target gene networks within the microenvironment. Nevertheless, there is currently a lack of studies directly comparing the dynamic changes in the target gene profile of miR-146a across different degeneration stages within the same longitudinal animal model. Existing evidence indicates that this phenomenon more closely resembles a “target bias“ emerging within specific microenvironments, rather than an absolute “functional reversal“ of the miRNA itself.

These findings support CDMSM by indicating that inflammatory intensity and oxidative-stress state may redirect miRNA-associated regulatory output. Under moderate inflammatory activation, a miRNA may buffer TRAF6/IRAK1/NF-κB signaling, whereas under chronic oxidative injury the same or related miRNA network may become associated with impaired antioxidant defense, mitochondrial dysfunction, or cell damage; cytokine-driven inflammatory amplification is also a well-recognized component of disc degeneration (Risbud and Shapiro, 2014). Direct longitudinal validation is still required to determine whether this represents true target switching or model-dependent divergence (Lv et al., 2017; Gao et al., 2025).

## 3 miRNAs and programmed cell death

### 3.1 Apoptosis

Evidence demonstrates that under early stress conditions characterized by mild hypoxia and nutrient deprivation, miR-155–3p can effectively induce autophagy by inhibiting KDM3A expression and regulating HIF-1α-related pathways, exerting a protective role in maintaining matrix homeostasis and promoting NP cell survival (Zhou et al., 2021). With the progression of degeneration or persistent hypoxia, the HIF-1α-related pathway becomes the core mechanism for autophagic compensation in NP cells, impacting cell survival (He et al., 2021). Under this extreme pathological background, if miR-155–3p continuously exerts excessive inhibition on the HIF-1α-related pathway, it may directly block this critical endogenous adaptive channel, diminishing the cells’ stress resistance capability and increasing the risk of cell death. As pointed out in recent literature, the biological outcomes of key stress molecules such as HIF-1α are highly dependent on the dynamic evolution of local oxygen tension and the microenvironment (Li et al., 2021). It is worth noting that the aforementioned inference is based on an indirect comparison between two independent studies—the protective effect of miR-155–3p in an early hypoxia model versus its potential detrimental effect under late-stage persistent hypoxia has not yet been simultaneously observed within the same longitudinal model. Therefore, this “functional transition“ is currently more accurately characterized as a hypothesis awaiting validation, rather than an established mechanism.

This framework maintains a rigorous distinction between established experimental evidence and hypothesis-driven extrapolation. The protective role of miR-155–3p under defined stress conditions is supported by experimental data, whereas the proposed loss of benefit or detrimental effect under persistent late-stage hypoxia should be regarded as a testable prediction requiring same-model, time-resolved validation (Zhou et al., 2021; He et al., 2021).

### 3.2 Pyroptosis and ferroptosis

In the regulation of pyroptosis, miR-128-3p can effectively target and inhibit the TRAF6-NF-κB signaling axis through an exosome-mediated manner, thereby reducing the abnormal activation of the NLRP3 inflammasome and decreasing cell pyroptosis (Li et al., 2024). However, when the intensity of local inflammatory stimulation exceeds the cell’s endogenous compensatory limits, the degenerative microenvironment causes a progressive decline in the expression level of miR-128-3p, which may ultimately render this endogenous protective mechanism completely ineffective (Li et al., 2024).

Regarding ferroptosis, studies have confirmed that miR-19b-3p can directly target ACSL4 to inhibit the lipid peroxidation cascade, thereby maintaining intracellular iron metabolism homeostasis under basal or mild stress conditions (Feng et al., 2025). However, as degeneration advances to severe stages, widespread transcriptomic alterations trigger a global remodeling of the ceRNA network (Zhu et al., 2019). Against this complex backdrop, a substantial number of aberrantly expressed lncRNAs or circRNAs may exert a “sponge“ effect, massively sequestering and consuming miR-19b-3p, leading to a sharp decrease in its free effective concentration, and consequently weakening or even reversing its inhibitory effect on ferroptosis (Table 1).

**TABLE 1:** This table summarizes the functional heterogeneity of specific microRNAs based on their microenvironmental contexts in IDD models. “Context A” and “Context B” highlight the distinct biological outcomes when the identical miRNA is subjected to varying disease stages, mechanical loads, or inflammatory intensities. The evidence underscores the necessity of matching therapeutic interventions with the dynamic transcriptomic and microenvironmental states of the disc.

MicroRNA	Outcome in context A	Specific conditions (Context A)	Outcome in context B	Specific conditions (Context B)	Proposed mechanism/Core targets	Evidence level/Interpretation
miR-494	Pro-apoptotic	Acute inflammatory stimulation	Enhanced matrix degradation	Severely degenerated samples	Pathological environment-driven transition/Positive correlation with decreased SOX9	Direct *in vitro* and human degenerative-sample evidence; context comparison remains cross-sectional
miR-365	Matrix homeostasis preservation and anti-apoptotic	Basal/moderate stress or physiological pressure (0.5–2 MPa)	Accelerated degeneration and ECM destruction	Pathological loading or high hydrostatic pressure (>3 MPa)	Mechanosensitive target network remodeling/Targeting EFNA3	Direct mechanobiology and NP-cell evidence; target network may differ by cell type and loading regimen
miR-146a	Anti-inflammatory and ECM preservation	Early inflammatory models (e.g., IL-1β, TNF-α upregulation)	Exacerbated oxidative stress and cellular damage	Progressive deterioration/High oxidative stress	Target switching/Bias from TRAF6/IRAK1 (context A) to SOD2 (context B)	Direct anti-inflammatory target evidence plus oxidative-stress target evidence; switching requires longitudinal validation
miR-155–3p	Autophagy induction and cell survival	Early stress, mild hypoxia, and nutrient deprivation	Diminished stress resistance and promoted cell death	Persistent late-stage hypoxia	Excessive inhibition of HIF-1α-related adaptive pathways/Targeting KDM3A	Direct stress-adaptation evidence; late-stage detrimental interpretation remains hypothesis-generating
miR-128–3p	Anti-pyroptosis	Endogenous compensatory limits	Loss of protective mechanism	Severe/overwhelming local inflammatory stimulation	Expression decline/Targeting the TRAF6-NF-κB signaling axis	Direct exosomal anti-pyroptosis evidence; loss of protection under severe inflammation is inferred from inflammatory context
miR-19b-3p	Anti-ferroptosis and iron homeostasis	Basal or mild stress conditions	Weakened or reversed anti-ferroptosis effect	Severe stages of degeneration	Global ceRNA network remodeling (sponged by lncRNAs/circRNAs)/Targeting ACSL4	Direct ACSL4/ferroptosis evidence; ceRNA-sponging effect in late IDD is network-inferred and requires validation

Abbreviations: IDD, intervertebral disc degeneration; ECM, extracellular matrix.

The examples in this section indicate that miRNA-related regulation of programmed cell death is likely to depend on inflammatory burden, iron metabolism, oxidative stress, and ceRNA competition. While the mechanisms explored in Section 3 are fundamental to disc pathology, the current analysis prioritizes validated pathways over indirect comparisons between independent studies (Li et al., 2024; Feng et al., 2025).

The additional evidence-level column distinguishes direct experimental support from indirect, cross-sectional, or network-inferred interpretation. This distinction is important because CDMSM is intended to generate testable predictions, not to overstate the certainty of functional switching where longitudinal evidence is unavailable.

## 4 Mechanisms underlying the functional heterogeneity of miRNAs

Although the regulatory roles of miRNAs in IDD have been widely reported, the phenomenon wherein the same miRNA exhibits diametrically opposed functions across different studies remains a contradiction that needs to be clarified (Chen et al., 2023). The discrepancies in conclusions among different studies, apart from inconsistent experimental conditions, may stem fundamentally from differences in the molecular regulatory networks in which the miRNAs are situated.

Importantly, apparent discrepancies in miRNA function should not automatically be interpreted as biological switching. As emphasized in previous bibliometric and methodological analyses of IDD miRNA research (Chen et al., 2023), differences in experimental model, species, tissue source, cell passage, degeneration grade, mechanical regimen, inflammatory stimulus, intervention dose, transfection efficiency, time point, and endpoint selection may independently produce inconsistent conclusions. CDMSM should therefore be considered valid only if context-dependent differences persist after these methodological variables are controlled.

### 4.1 Hierarchical interpretation of CDMSM

To improve mechanistic integration, CDMSM can be interpreted as a hierarchical regulatory architecture rather than a simple linear model of miRNA functional transition. Within this framework, context-dependent miRNA behavior emerges from interactions among multiple biological layers during IDD progression. At the upstream level, degeneration-associated mechanical loading, inflammatory stimulation, oxidative stress, hypoxia/acidity, metabolic imbalance, and degeneration stage act as primary contextual inputs. These factors reshape the transcriptomic and ceRNA landscape of disc cells, thereby altering miRNA effective concentration, target accessibility, and pathway dominance. As network remodeling progresses, the same miRNA may become preferentially associated with different downstream signaling outputs under distinct pathological contexts, including ECM preservation, inflammatory buffering, autophagy, ferroptosis, pyroptosis, or matrix-catabolic programs.

Accordingly, CDMSM can be conceptually organized into five hierarchical layers: contextual inputs, including mechanical loading, inflammation, oxidative stress, hypoxia/acidity, and degeneration stage; network remodeling, including ceRNA competition, transcriptomic reprogramming, and altered target abundance or accessibility; pathway dominance, involving NF-κB, MAPK, HIF-1α, YAP/TAZ, autophagy, ferroptosis, and pyroptosis-related signaling; phenotypic output, including ECM maintenance or degradation, adaptive survival, senescence, inflammation, and cell death; and therapeutic windows, including stage-specific responsiveness to miRNA supplementation, antagomirs, engineered exosomes, and smart-responsive biomaterials.

Importantly, this hierarchical interpretation does not imply that deterministic functional switching has already been experimentally proven. Rather, it provides a systems-level conceptual framework for generating testable hypotheses regarding how biological context may reshape miRNA-associated regulatory output during IDD progression (Cazzanelli and Wuertz-Kozak, 2020; Chen et al., 2023).

### 4.2 Stage-dependent functional transition

IDD is a progressive process transitioning gradually from adaptive stress to structural instability. Based on current cross-sectional studies, we propose that miRNAs may exert differential effects by regulating distinct molecular networks across various stages of degeneration. In the early stages of degeneration or under moderate stress conditions, miRNA-mediated regulation may favor cellular adaptive responses, such as promoting autophagy or restricting inflammation spread; conversely, as the severity of degeneration increases, changes in the downstream target gene profiles and the dominance of signaling pathways cause the overall function of the miRNA to become associated with pro-degenerative processes (Liu et al., 2023). Nevertheless, there is currently a lack of longitudinal studies to directly validate this stage-specific functional remodeling process within the same model.

Within the CDMSM framework, stage dependence is defined as a falsifiable hypothesis rather than a proven linear transition. A rigorous test would require longitudinal sampling within the same degeneration model, direct measurement of miRNA-bound targets or target accessibility, and functional perturbation at multiple stages. Failure to observe reproducible stage-related changes in miRNA target bias under controlled conditions would challenge CDMSM.

### 4.3 Remodeling of the ceRNA network

The actual regulatory effect of a miRNA is largely influenced by the ceRNA network, which can alter the effective action intensity of the miRNA. The progression of IDD is generally accompanied by a remodeling of the lncRNA expression profile; when specific lncRNAs act as sponges to sequester large amounts of a miRNA, a phenomenon may occur where the effective function of the miRNA significantly decreases even if its total amount remains constant (Yu et al., 2022). Therefore, we posit that this might be a key reason why identical levels of a miRNA produce diverse phenotypic outcomes in different samples.

Mechanistically, ceRNA remodeling may alter miRNA function without changing total miRNA abundance. Increased lncRNA or circRNA abundance can reduce the free effective concentration of a miRNA, while changes in mRNA target abundance can redirect the same miRNA toward different pathways. Therefore, future studies should combine total miRNA quantification with target-validation assays, RNA interaction analyses, and network-level modeling (Zhu et al., 2019; Yu et al., 2022).

### 4.4 Differences in signal sensitivity induced by mechano-biological coupling

Biomechanical studies demonstrate that intervertebral disc cells possess high mechanosensitivity. Experiments have indicated that cyclic stretching (simulating physiological activity) and hydrostatic pressure (simulating pathological loading) can induce distinctly different miRNA expression profiles and signaling states. For instance, mechanical loading can induce the upregulation of miR-155-5p and activate the MAPK pathway, whereas the function of miR-155-5p is completely different under resting conditions (Cazzanelli et al., 2024). Consequently, we hypothesize that this unique mechanobiological dependence is also a major cause of the inconsistencies in miRNA functions.

Mechanical context is not merely a background variable in IDD; it is a primary determinant of disc-cell phenotype and miRNA-network output. Physiological cyclic loading may support adaptive matrix turnover, whereas sustained overload, abnormal hydrostatic pressure, or altered substrate stiffness may activate MAPK, NF-κB, HIF-1α, integrin, and YAP/TAZ-related mechanotransduction pathways. Mechanosensitive transcriptional coactivators MRTF-A and YAP/TAZ have been shown to regulate nucleus pulposus cell phenotype through cell shape (Fearing et al., 2019), supporting the idea that miRNA effects should be interpreted together with mechanical state and cell morphology (Chan et al., 2011; Cazzanelli et al., 2024).

### 4.5 Systemic integration of feedback loops

In complex biological systems, cellular functions are typically not determined by a single molecule, but are collectively regulated by multiple signaling pathways and their feedback loops. Studies have discovered that miRNAs are frequently embedded within intricate feedback loops of core pathways such as NF-κB and SIRT1 (Ji et al., 2018). Therefore, the basal state (activated or inhibited) of the core pathways affects the trajectory of the feedback loops. When the background network undergoes remodeling, the intervention of a miRNA may not simply reflect its intrinsic role, but could generate disparate systemic effects within the loop, such as amplifying signals or buffering oscillations.

This feedback-loop perspective also helps reconcile apparently divergent miRNA effects. For example, a miRNA that dampens NF-κB activation under moderate inflammatory stimulation may become insufficient when cytokine amplification exceeds a threshold, while a miRNA that supports autophagic adaptation under early stress may become maladaptive if it suppresses a pathway required for late-stage survival. These statements remain mechanistic hypotheses unless tested by pathway-specific perturbation.

### 4.6 Functional differences obscured by cellular heterogeneity

Previous studies have often yielded conflicting conclusions regarding miRNA functions, which is highly likely because traditional bulk tissue sequencing overlooks the complex cellular heterogeneity within the microenvironment. Evidence from single-cell RNA sequencing (scRNA-seq) suggests that the “reversal” of miRNA functions observed in degenerated tissues may originate from the dynamic succession of dominant cell subpopulations. On the one hand, differences in target gene abundance among various functional subpopulations can determine the final effect output of the same miRNA; on the other hand, immune cells infiltrating late-stage degenerated tissues carry high abundances of miRNAs, which can easily cause signal “contamination” in mixed samples, leading to the misinterpretation of exogenous interference as endogenous pathological changes in NP cells. Furthermore, ceRNA regulatory networks have been proven to possess high cell specificity, implying that “averaged” analyses lacking subpopulation resolution are a critical factor leading to off-target therapeutic strategies; hence, future research urgently needs to incorporate spatiotemporal transcriptomic technologies to precisely dissect molecular regulatory networks within specific pathological subpopulations.

Bulk-tissue measurements are particularly vulnerable to this problem because late-stage discs may contain altered proportions of NP-like cells, fibroblast-like cells, endplate-derived cells, senescent cells, and immune cells. Single-cell and spatial transcriptomic approaches are therefore needed to determine whether an observed miRNA change reflects true functional switching within the same cell population or a shift in the dominant cell population being sampled.

**FIGURE 2:**
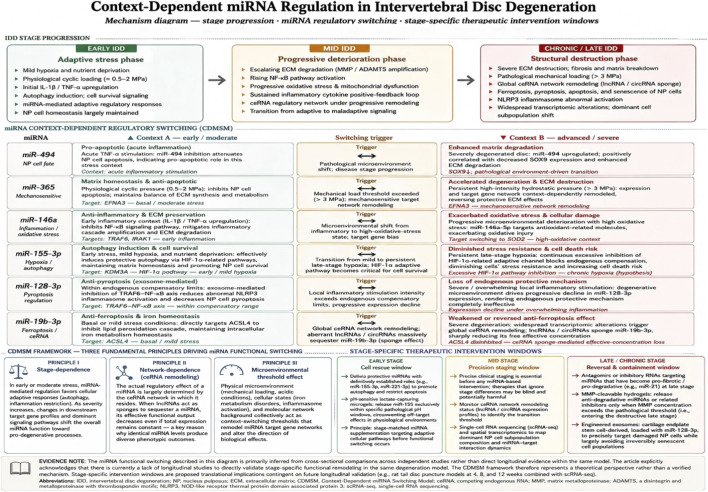
Proposed stage-dependent miRNA regulatory switching and therapeutic windows in IDD. In early-stage IDD, moderate stress and partial matrix preservation may allow selected miRNAs to support adaptive autophagy, ECM maintenance, and inflammatory buffering. During intermediate degeneration, mechanical overload, inflammatory amplification, oxidative stress, hypoxia/acidity, and ceRNA-network remodeling may alter miRNA target accessibility and effective concentration, leading to mixed or context-dependent biological outputs. In chronic/late-stage IDD, severe ECM disruption, senescence, programmed cell death, fibrosis, and cellular heterogeneity may bias miRNA networks toward catabolic or maladaptive outcomes. The CDMSM framework predicts that therapeutic strategies should be matched to disease stage, with protective miRNA supplementation favored in early adaptive contexts, combined network modulation in intermediate disease, and inhibitory or microenvironment-responsive strategies in late-stage degeneration.

As established in the hierarchical architecture above, degeneration-associated inputs ultimately bias miRNA networks toward distinct phenotypic outputs. This layered integration provides the mechanistic basis for stage-specific therapeutic windows and microenvironment-responsive intervention strategies (Figure 2).

## 5 Engineered delivery strategies

Given the highly context-dependent nature of miRNA functions discussed above, particularly the potential functional reversal of the same molecule between early (protective) and late (pathogenic) stages of degeneration, traditional strategies of direct supplementation or inhibition not only have questionable efficacy but may even exacerbate pathological damage due to inappropriate intervention timing. Therefore, the core of future miRNA therapies lies in achieving “stage matching” and “microenvironment-responsive release”.

To integrate translational strategies within the CDMSM framework, therapeutic implications are categorized according to stage-specific intervention windows. The central translational implication is that the same miRNA intervention may be beneficial, ineffective, or harmful depending on whether the disc is in an adaptive early stage, an inflammatory/mechanically overloaded intermediate stage, or a chronic late stage characterized by ECM collapse, senescence, and network remodeling.

### 5.1 Staged intervention

Before designing a delivery system, the principle of “staged intervention” must be considered. For cells exhibiting only mild damage in the early stage, miRNAs with definitively protective roles (e.g., anti-apoptotic, pro-autophagic), such as miR-155-3p or miR-221-3p, should be selected for delivery whenever possible to achieve “rescue” (Xu et al., 2025). For the late-stage microenvironment dominated by fibrosis and matrix breakdown, if certain molecules have transformed into pathogenic factors (such as the pro-fibrotic effect of miR-21), the strategy should be reversed to deliver their antagomirs or inhibitory RNAs targeting degrading enzymes. Clinical translation must be founded upon precise staging; miRNA therapies that ignore stage differences may be blind and potentially harmful.

Accordingly, early-stage disease may be more suitable for protective miRNA supplementation or exosome-mediated rescue, intermediate disease may require combined anti-inflammatory and anti-catabolic network modulation, and late-stage disease may require antagomirs, miRNA sponges, anti-fibrotic strategies, or biomaterials that release cargo only when MMP, ROS, pH, or inflammatory thresholds are reached (Ji et al., 2018; Guo et al., 2025).

### 5.2 Engineered exosomes

Exosomes are the most commonly utilized tools to achieve precise delivery at the cellular level. To address the issue of disparate responses to the same miRNA among different cell subpopulations, exosomes require engineered modifications. For instance, by utilizing cartilage endplate stem cell-derived exosomes loaded with protective miRNAs (such as miR-128-3p), their inherently carried signaling networks can function synergistically and precisely target damaged NP cells, thereby largely avoiding non-specific entry into irreversibly senescent cell populations and the subsequent induction of side effects.

Within CDMSM, engineered exosomes are most rational when the target disc still contains repair-responsive cell populations. Their value may depend not only on cargo selection but also on donor-cell source, loading efficiency, recipient-cell state, and whether the delivered miRNA restores a protective network rather than entering a maladaptive late-stage target landscape (Li et al., 2022; Li et al., 2024).

### 5.3 Smart responsive hydrogels

Targeting the “threshold effect” of miRNA functions, a hydrogel capable of sensing microenvironmental intensity can be designed. For example, a hydrogel containing MMP cleavage sites can be constructed. Only when the MMP concentration in the microenvironment exceeds a pathological threshold (i.e., entering the destructive late stage) will the hydrogel degrade and release anti-degradative miRNAs or related inhibitors. Furthermore, the acidic environment of the degenerated disc can be leveraged to design pH-sensitive linkers (e.g., lactate-capturing microgels delivering miR-155) (Guo et al., 2025), and ECM-mimetic dynamic hydrogels delivering nucleus pulposus progenitor cell-derived exosomes have also been reported (Wang et al., 2025), ensuring that the drug is released exclusively within a specific pathological pH window, thereby circumventing off-target effects in physiological or extremely pathological environments.

Smart biomaterials are directly compatible with the threshold component of CDMSM. MMP-cleavable, ROS-responsive, or pH-sensitive systems can be designed so that miRNA mimics or inhibitors are released only under defined pathological conditions. This strategy may reduce the risk of exposing early adaptive tissues to late-stage inhibitory therapy, or exposing chronically degenerated tissues to miRNAs that are protective only in early-stage contexts (Guo et al., 2025; Wang et al., 2025).

### 5.4 Translational challenges

We note a common evasive tendency in existing research: almost no team has reported “intervention failure” data. Which specific cell subpopulations experience off-target effects, what the frequency of occurrence is, and whether the consistency of miRNA loading across preparation batches meets clinical-grade standards—these issues are rarely addressed in the majority of proof-of-concept studies. Until these fundamental questions are answered, discussing a timeline for clinical translation may be premature.

A further translational challenge is patient stratification. Before clinical translation, miRNA therapy will require practical biomarkers that identify degeneration stage, inflammatory intensity, oxidative burden, and residual repair capacity. Imaging grade alone is unlikely to capture the molecular context required for CDMSM-guided treatment selection.

### 5.5 Testable predictions and experimental validation of CDMSM

To transition CDMSM from a conceptual framework to a clinically actionable and scientifically testable model, we propose the following mechanistic and falsifiable predictions.

1. The functional targets of specific miRNAs (e.g., miR-365 or miR-155–3p) will exhibit a stage-dependent shift from anabolic pathways (e.g., ECM synthesis and cellular homeostasis) to catabolic and inflammatory pathways (e.g., NF-κB activation, matrix degradation, and ferroptosis) during the progression from early to late-stage IDD. Failure to observe such directional switching under controlled longitudinal conditions would challenge the validity of CDMSM.
2. Microenvironmental stressors, including mechanical overload and inflammatory bursts, will dynamically remodel the ceRNA network topology, resulting in measurable alterations in miRNA effective concentration and target accessibility within specific nucleus pulposus cell subpopulations.

To rigorously validate these predictions, longitudinal experimental designs are essential. We recommend time-resolved animal models (e.g., rat tail disc puncture models at 4, 8, and 12 weeks), combined with spatial transcriptomics (e.g., scRNA-seq) to map the spatiotemporal dynamics of miRNA–target interactions. Importantly, these approaches should be integrated with functional perturbation experiments to determine whether observed network reconfigurations causally drive shifts in cell fate decisions.

Future validation should also incorporate comparative models, including naturally occurring canine IDD, while accounting for species-specific differences in notochordal-cell persistence and disc biomechanics (Bergknut et al., 2012; Hunter et al., 2004). Controlled mechanobiology platforms should be used to vary loading intensity, duration, oxygen tension, acidity, and inflammatory stimulation in a factorial design so that biological context can be separated from methodological noise.

Finally, validation of CDMSM should extend to therapeutic testing: microenvironment-responsive delivery systems, such as stage-specific engineered exosomes and MMP-cleavable hydrogels, are expected to demonstrate differential efficacy depending on disease stage, thereby providing a functional readout of the model’s predictive power (Ji et al., 2018; Guo et al., 2025; Wang et al., 2025).

## 6 Conclusion and perspectives

Existing evidence suggests that the roles of miRNAs in IDD are not rigidly manifested as protective or pathogenic, but are more likely dependent on the dynamic molecular regulatory environment in which they reside. The functional discrepancies reported by different studies may reflect variations in the molecular environment of the disc across different stages of degeneration or under varying mechanical and inflammatory conditions. Although there is currently a lack of longitudinal evidence to directly validate the stage-specific functional remodeling of miRNAs, re-examining these research findings from a context-dependent perspective may help elucidate the seemingly contradictory observations in previous literature, thereby providing a theoretical framework for the rational design of miRNA-targeted therapeutic strategies. The “context dependence” framework proposed in this review currently remains a perspective rather than a verified theory. For it to yield genuine clinical guiding value, researchers need to address a specific and challenging question: Does the effective target gene profile of the same miRNA genuinely undergo reproducible, systemic remodeling at different time points within the same degeneration model? Until this question is answered affirmatively, therapeutic designs based on “stage matching” remain conjectural (Cazzanelli and Wuertz-Kozak, 2020; Chen et al., 2023).

In summary, the CDMSM provides a specialized IDD-focused framework that systematically applies context-dependent regulation to the dynamic, mechanically loaded, and inflammatory environment of the disc, rather than claiming this principle is unique to IDD. The framework should be considered useful only to the extent that its predictions can be verified by longitudinal, spatially resolved, and functionally perturbative studies.

## Data Availability

The original contributions presented in the study are included in the article/supplementary material, further inquiries can be directed to the corresponding author.
